# A new method to deliver supraclavicular radiation in breast radiotherapy for lung sparing

**DOI:** 10.1120/jacmp.v12i3.3374

**Published:** 2011-04-18

**Authors:** Bo Yang, Zheng Dong, Mu‐Han Lin, C‐M Ma

**Affiliations:** ^1^ Department of Radiation Oncology the Fourth Affiliated Hospital of Suzhou University Wuxi China; ^2^ Department of Radiation Oncology Fox Chase Cancer Center Philadelphia USA

**Keywords:** breast cancer, radiotherapy, supraclavicular irradiation, radiation pneumonia

## Abstract

Due to the angulation of the breast board used for tangential breast irradiation, additional normal lung tissues are included in the supraclavicular field. This work investigates a method to reduce the lung volume and dose delivered during supraclavicular irradiation for breast cancer. Ten patients included for this retrospective study received chest wall and supraclavicular irradiation following radical surgery or breast‐conserving surgery. Three‐dimensional conformal radiation therapy plans were generated using the CMS XiO treatment planning system. The clinical target volume (CTV) of the supraclavicular irradiation is defined as the subcutaneous tissues from 0.5 cm under the anterior skin surface to a 3 cm depth. Only the ipsilateral lung is defined as the organ at risk. In the new method, the couch is rotated 90° and the supraclavicular field is tilted to maintain a normal incident angle to the breast board rather than the couch surface to spare more normal lung tissues. The absolute volume of the ipsilateral lung irradiated, and the volumes of lung tissues receiving 5 Gy and 20 Gy $(V_{5}$ and $V_{20})$ are analyzed. The new method can reduce the lung volume irradiated by the supraclavicular field significantly. For the ten patients investigated, only 5.3% of the ipsilateral lung is irradiated with the new method, while 14.9% of the ipsilateral lung is irradiated using the conventional method. Compared with the conventional method, the new method reduces $V_{5}$ by 53.6% and $V_{20}$ by 59.0%. Our new method does not alter the patient positioning for breast treatment but rotates the couch to deliver a tilted supraclavicular field to maintain adequate CTV coverage and spare more normal lung tissues. The results of this study demonstrated that our new method is effective, and that the reduction of normal lung tissue volume in the field is significant.

PACS number: 87.55.D‐

## I. INTRODUCTION

Breast cancer is the most common malignancy among women in China. For decades, a comprehensive treatment strategy has been applied for breast cancer patients, which includes radical or breast‐conserving surgery, radiotherapy, chemotherapy, endocrine therapy, and immunization therapy.^(^ ^1^ ^–^ ^2^ ^)^ Since local recurrence remains a primary factor leading to death in patients, radiation therapy has become an important component of the comprehensive treatment to reduce local recurrence.^(^ ^3^ ^–^ ^5^ ^)^

Generally, radiotherapy after radical mastectomy or breast‐conserving surgery routinely includes the chest wall. For maxillary lymph node‐positive patients, the supraclavicular region should also be regularly irradiated, because the invasion rate of the supraclavicular area is up to 20% ~ 40%, with 25% involving the supraclavicular area in relapsed patients.^(6)^ For patients receiving irradiation to both the chest wall and the supraclavicular area, the irradiation fields are generally divided into two groups by the baseline of the lower edge of the subclavian head: one pair of tangential beams to cover the chest wall and one anterior‐posterior beam to cover the upper supraclavicular area.

Cohesion of the upper and lower groups can be performed by the quarter‐field irradiation technique with the same irradiation center, or by the symmetric fields technique with an adequate distance between the fields to avoid hot or cold spots.^(^ ^7^ ^–^ ^8^ ^)^

**Figure 1:**
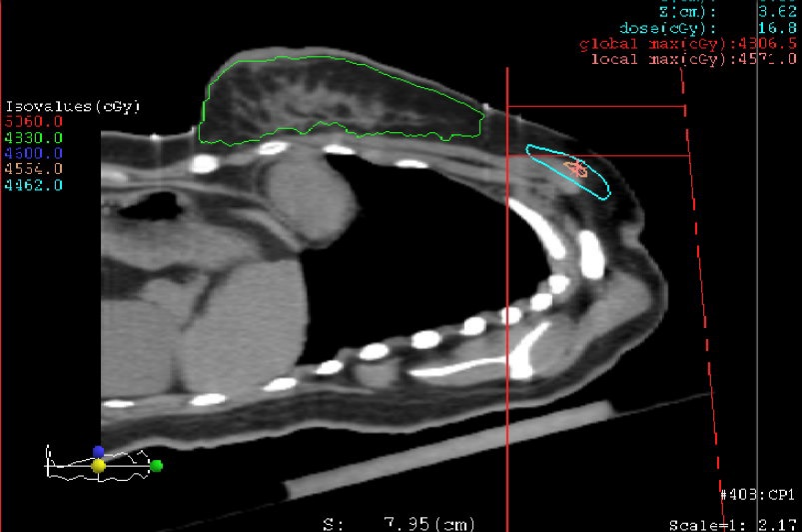
Conventional treatment setup for supraclavicular irradiation.

To ensure setup reproducibility and the cohesion of the upper and lower groups, the breast board is usually used to lift the upper body of the patient at a certain angle^(^ ^9^ ^–^ ^10^ ^)^ without rotating the collimator. Both the supraclavicular field and the tangential beams are delivered under the same conditions, as shown in Fig. 1, to make sure that the two tangential fields match the supraclavicular field at the cut line.

The reference depth of the supraclavicular area is defined as 3 cm. In consideration of the coverage or sparing of the skin, generally 6 MV X‐rays and electrons are mixed to deliver 46 Gy to the treatment target. In Fig. 1, we can easily see that because of the elevation of the upper body of the patient and the cone shape of the irradiation beam, the irradiated volume of lung tissue in the supraclavicular field by the conventional radiotherapy method can be significantly reduced compared with the vertical radiation method.

When patients are treated with supraclavicular irradiation, the radiation target is the subcutaneous tissue from 0.5 cm under the anterior skin surface to a 3 cm depth. Lung tissues need to be spared as much as possible. The incidence rate of radiation pneumonitis resulting from postoperative radiotherapy for breast cancer is 5% to 15%,^(11)^ and a dose of 20 Gy rarely leads to radiation pneumonitis. It is closely related to age, sex, radiation field size, dosage, fractionation, and whether the patient also received chemotherapy, as well as other factors.^(^ ^12^ ^–^ ^15^ ^)^ Radiation pneumonitis is a major complication of radiotherapy for our breast cancer patients, especially for those who have received chemotherapy.^(16)^ The six‐month post‐radiotherapy follow‐up for the 264 patients who received postoperative radiotherapy indicates that the occurrence of radiation pneumonitis is 11% (29/264). Twenty‐one percent of the 29 patients are level III and IV radiation pneumonitis, which significantly affects their quality of life. Therefore, efforts are being made to reduce the volume and dose delivered to lung tissues for breast cancer treatment.

## II. MATERIALS AND METHODS

**Figure 2:**
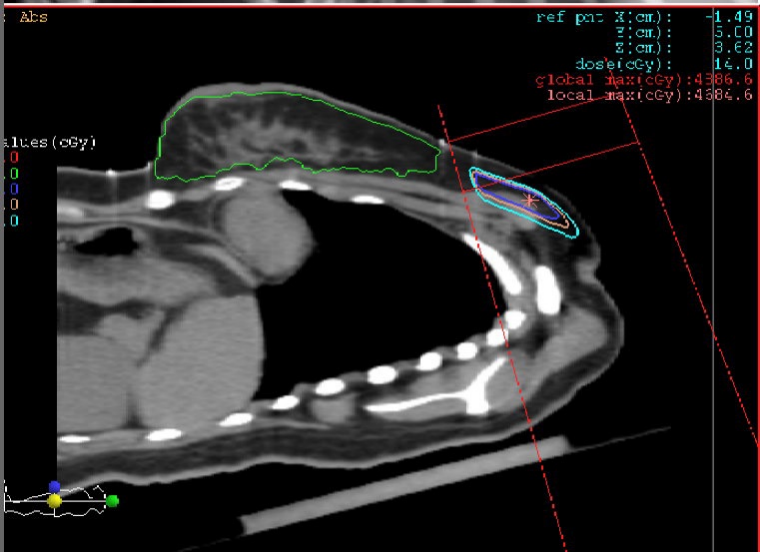
Modified treatment setup for supraclavicular irradiation.

This paper investigates an improved treatment setup for breast patients in order to reduce the lung volume irradiated by the supraclavicular field. This is achieved by rotating the couch 90° and tilting the supraclavicular field to maintain a vertical incident angle to the bottom of the breast board, as shown in Fig. 2. It is anticipated that the reduction of the volume and dose delivered to lung tissues will decrease the incidence of radiation pneumonitis. This work will quantify the lung volume and dose reduction with the new setup method in comparison with the conventional treatment setup.

Ten patients were included for this retrospective study, each of whom had received conventional chest wall and supraclavicular irradiation following radical surgery or breast‐conserving surgery.

CT and MRI scans were performed for each patient in the supine position with the breast board, abducting the ipsilateral upper extremity 90°. The scanned area was expanded from the mastoid process to 5 cm under the contralateral breast fold with a 0.3 cm slice spacing. The CT and MR images were fused on the three‐dimensional treatment planning system.

### A. Equipment

Varian 23ex linear accelerator with a 120 multileaf collimator; Varian Eclipse treatment planning system; Varian ARIA record and verify system (Varian Medical Systems, Palo Alto, CA); Elekta/CMS XiO treatment planning system (St. Louis, MO); Picker PQS Single‐slice spiral CT (Cleveland, OH) and MED‐TEC MT350 breast board (Marietta, Georgia).

### B. Definition of the treatment target and organ at risk

The clinical target volume (CTV) for the supraclavicular irradiation was defined as the subcutaneous tissues from 0.5 cm under the anterior skin surface to a 3 cm depth. The inner border was the contralateral edge of the sternocleidomastoid muscle, the outer border was the inner edge of the humeral head, the upper border was the cricothyroid membrane, and the lower edge was the lower border of the clavicle. The planning target volume (PTV) was the CTV plus a 1 cm margin in the superior and inferior directions, and a 0.7 cm margin laterally. No extra margin was used for the anterior‐posterior direction. Only the ipsilateral lung was defined as the organ at risk.

### C. Treatment planning design

Three‐dimensional conformal radiation therapy plans were generated using the XiO treatment planning system (CMS, St. Louis, MO). Treatment plans were generated for the conventional setup for supraclavicular irradiation, as shown in Fig. 1, which were used for the patient treatment. Our modified method required rotation of the couch 90°, and then rotation of the gantry angles to ensure the supraclavicular beam incident normally to the breast board, as shown in Fig. 2. The gantry rotation angle, therefore, depended on the breast board angle, which is typically 16°–18°. All plans used 6 MV photon beams with a fixed source‐to‐surface distance. There was a small (about 3 mm) overlap of the supraclavicular field with the tangential field border on the skin surface. The gantry rotation and the field overlap resulted in some hot spots in the tangential field and some cold spots in the supraclavicular field, which were carefully adjusted during treatment planning to minimize the hot spots and cold spots (generally < 10%). The dose prescription was PTV ≥ 46 Gy at 2 Gy/fractions for 23 fractions.^(^ ^11^ ^,^ ^17^ ^)^

### D. Evaluation criteria

The absolute volume of the ipsilateral lung irradiated, the volumes of lung tissues receiving 5 Gy and 20 Gy $(V_{5}$ and $V_{20}$), and the maximum and minimum lung doses were analyzed.

### E. Statistical analysis

Data were analyzed using the SPSS13.0 software package.

## III. RESULTS

The volume and dose of lung tissues irradiated by the conventional setup and the new method are shown in Table 1 for the ten patients investigated.

**Table 1:** Comparison of lung tissues irradiated by two different setup methods in postoperative supraclavicular radiation therapy for breast cancer.

*Ipsilateral Lung Cases*		*Conventional Method Volume of Lung*			*New Method Volume of Lung*		
*Volume (cc)*		*Irradiated (cc)*	$V_{5}(\%)$	$V_{20}(\%)$	Irradiated (cc)	$V_{5}(\%)$	$V_{20}(\%)$
1	741.91	125.46	23.71	19.14	35.93	8.77	5.21
2	936.85	180.47	24.90	21.01	78.90	13.16	10.13
3	944.59	98.81	15.39	11.65	21.70	4.57	2.62
4	1000.18	148.85	21.98	18.41	52.85	12.76	9.84
5	1080.80	119.57	15.09	11.84	41.04	6.15	4.16
6	1169.40	153.22	9.77	5.54	38.89	5.11	2.17
7	1184.56	169.72	17.32	13.93	61.90	8.86	7.00
8	1223.93	200.64	21.19	16.96	64.75	8.26	5.71
9	1423.14	169.91	15.87	12.57	57.67	6.80	4.70
10	1565.82	316.87	27.11	23.24	159.74	14.79	11.76
Average	1127.12	168.35	19.23	15.43	61.34	8.92	6.33

Table I summarizes the irradiated ipsilateral lung volume and the $V^{5}$ and $V^{20}$ of the conventional method and the new method. The average/maximum irradiated volume of the conventional method is 168.4 cc/316.9 cc, which corresponds to 14.9%/20.2% of the total volume of the ipsilateral lung. Compared to the conventional method, the average/maximum irradiated lung volume of the new method is 61.34 cc/159.74 cc, which corresponds to 5.4 %/10.2% of the total volume of the ipsilateral lung.

The maximum and minimum lung doses received in postoperative supraclavicular radiation therapy for breast cancer using the conventional setup method is $D_{\text{max}}=41.33$ Gy and $D_{\text{min}}=6.70$ Gy, averaged over the ten patients. In comparison, $D_{\text{max}}=39$. 81 Gy and $D_{\text{min}}=2.87$ Gy using the new treatment setup.

**Figure 3:**
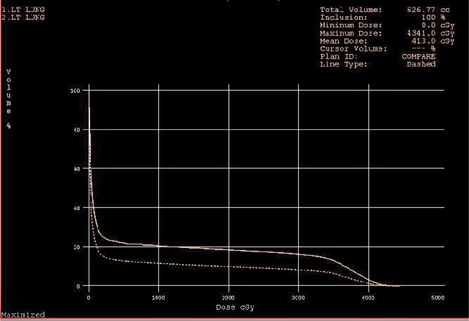
DVHs of the two methods.

The dose volume histograms (DVHs) of the ipsilateral lung for one patient using the two treatment setup methods are shown in Fig. 3. The solid line is the DVH of new method and the dotted line is the DVH of the conventional method.

**Figure 4:**
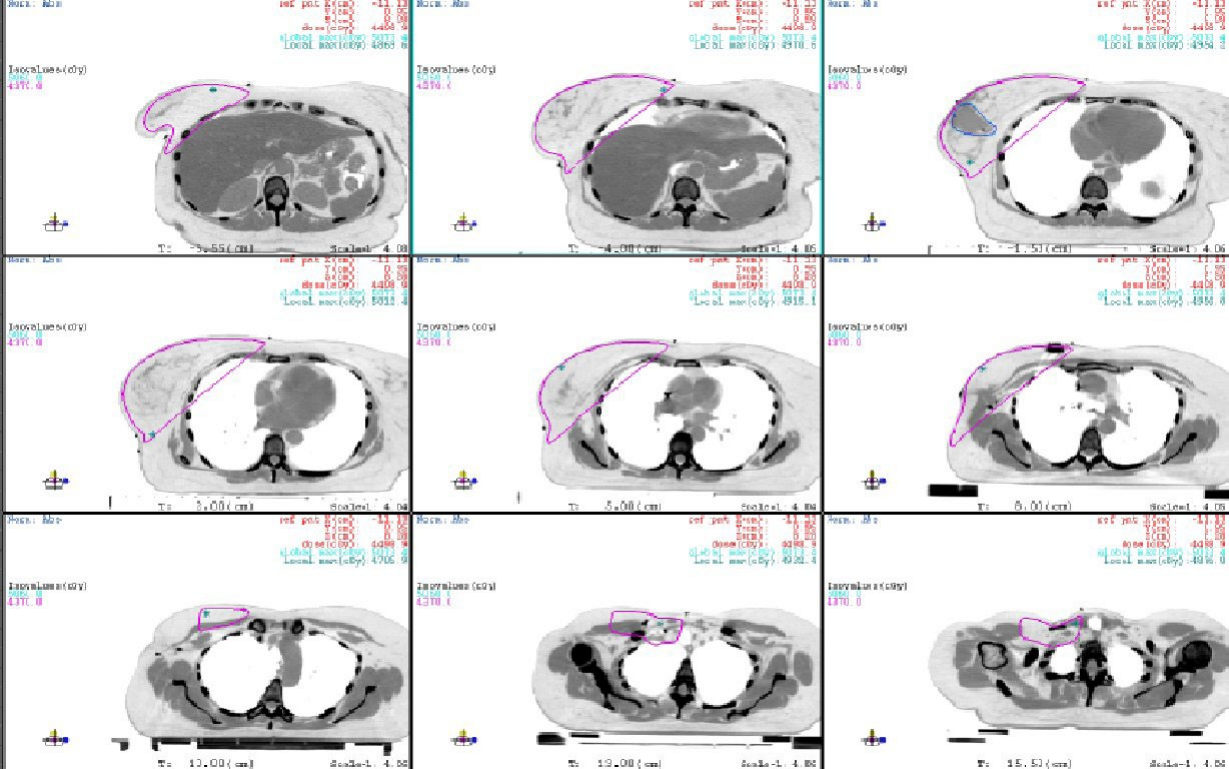
The 95% isodose curve of each dose plane of the new method.

Figure 4 shows the 95% isodose distributions of the new method. Overall, the coverage is comparable to the conventional method.

**Figure 5:**
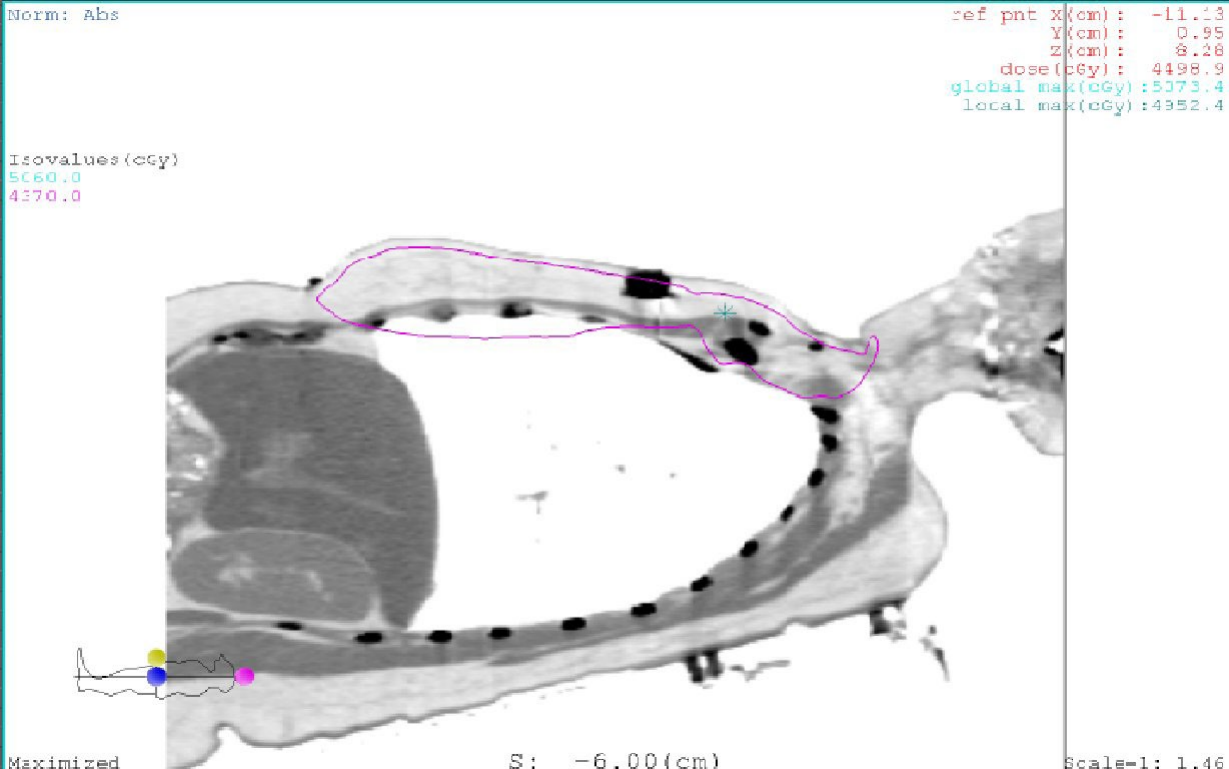
The 95% isodose curve at the central sagittal plane for the new method.

To investigate the dose distribution in the field junction region, the 95% isodose curve of the central sagittal plane is shown in Fig. 5. As shown in the figure, the CTV is fully covered by the 95% isodose curve.

## IV. DISCUSSION

Breast cancer is generally considered as a systemic disease, which seriously affects the health of women.^(^ ^1^ ^–^ ^2^ ^)^ Surgery is the primary choice for breast cancer treatment. Radical surgery is still the preferred treatment in China, and the application of breast‐conserving surgery has garnered significant interest in the last decade. Breast‐conserving surgery is regarded as safe and reliable without affecting the efficacy of treatment.^(^ ^18^ ^–^ ^21^ ^)^ However, the recurrence of breast cancer remains a primary factor leading to death. As a result, a comprehensive treatment strategy has been applied as the most effective treatment, which includes surgery, radiotherapy, chemotherapy, endocrine therapy, and immunotherapy.

Radiation therapy can significantly decrease the risk of regional recurrence, especially for patients who received breast‐conserving surgery. The regional recurrence was reduced by 75% when postoperative radiotherapy was performed, and by 65% for patients having radical surgery. Postoperative radiotherapy has become an important part of treatment for breast cancer.^(^ ^3^ ^–^ ^5^ ^)^ Treatment decisions depend on the location, size, histological type of the tumor, as well as auxiliary lymph node metastases. Generally, the chest wall should be irradiated, and the supraclavicular region should also be included when the auxiliary lymph nodes are positive. For patients who receive both chest wall and supraclavicular irradiation, a breast board is usually used to ensure the irradiation stability, the treatment setup reproducibility, and the cohesion of the tangential beams and the supraclavicular field.^(^ ^6^ ^,^ ^9^ ^,^ ^10^ ^)^ Due to the angulation of the breast board for tangential chest wall irradiation, the irradiated volume of lung tissue in the supraclavicular field using the conventional treatment setup is significant, compared to the method proposed in this work.

The most common acute adverse effects reported are radiation‐induced skin damage and radiation pneumonitis. Radiation pneumonitis is a lung injury that occurs when a large volume of normal lung tissue is irradiated or a large dose of radiation is administered. It is marked by acute exudative degeneration followed by chronic fibrosis.^(22)^ Its occurrence and severity are closely related to the radiation dose, the daily fraction, and the amount of lung exposed to certain doses of radiation. Certain chemotherapy or targeted agents may make the lung more sensitive to radiation pneumonitis, or may actually cause it independently. According to the clinical results from Zhongshan Hospital, Shanghai Medical University, Shanghai, China, radiation pneumonitis rarely occurs for lung doses less than 20 Gy in six weeks. When the lung dose was more than 40 Gy, radiation pneumonitis increased significantly, and it is certain to occur with lung doses exceeding 60 Gy.^(^ ^12^ ^–^ ^15^ ^)^ The consequences of radiation pneumonitis vary widely. Some patients appear asymptomatic, while others suffer from continuous cough; and in more serious cases, it can even lead to death.^(16)^

Significant efforts have been made to reduce the incidence rate of radiation pneumonitis. It has been suggested that cohesion of the chest wall and the supraclavicular area can be solved by the quarter‐field irradiation technique. It can avoid the hot or cold spots and also reduce the irradiated lung volume; the 90% isodose line is reduced by 0.9 cm compared to the total field irradiation. However, the lung tissues irradiated by the supraclavicular field can still be substantial. Others suggested that the irradiation of the chest wall and the supraclavicular area should be carried out in steps, or by increasing the treatment fractions with reduced fractional dose, or by making use of appropriate tissue‐equivalent bolus for chest wall irradiation which can reduce the irradiated dose to the anterior border of the lung tissues. Avoiding the synergy with chemotherapy and preventing the deterioration of radiation pneumonitis with regular observation are also suggested. They all have a certain effect on the prevention and reduction of radiation pneumonitis.^(^ ^17^ ^,^ ^23^ ^)^

As a severe complication of radiation therapy, radiation pneumonitis should be avoided as much as possible in clinical practice. For breast cancer patients who received postoperative radiotherapy, reducing the irradiated volume and dose of lung tissue may lead to the prevention and reduction of radiation pneumonia. According to our statistical analysis, the new method can reduce the lung volume irradiated by the supraclavicular field significantly. For the ten patients investigated, only 5.3% of the ipsilateral lung was irradiated, while 14.9% of the ipsilateral lung was irradiated using the conventional treatment setup. Compared with the conventional method, the new method reduced $V^{5}$ and $V^{20}$ by 53.6% and 59.0%, respectively.

Our results showed large differences in the lung volume irradiated (up to 316.9 cc in some patients), which is certainly of clinical significance. This may be related to the patient's body shape and lung geometry. Therefore, we should pay special attention to the individual differences during treatment planning. However, for the same patient, the new method can always reduce the volume and dose of the lung tissues irradiated.

The implementation of the new method is relatively simple. One only needs to rotate the couch by 90° and the treatment gantry according to the breast board angle, and also optimize the doses from the noncoplanar beams. It is important to select a proper overlap between the supraclavicular field and the tangential beams to minimize the hot spots in the tangential field and the cold spots in the supraclavicular field. Our results show that a 3 mm overlap on the skin surface (i.e., moving the supraclavicular field edge inferiorly by 3 mm) is generally adequate. The field‐in‐field technique can also be used for the tangential beams to minimize the hot and cold spots. It is possible to keep the hot and cold spots to within 10%, as demonstrated by our studies. Further clinical investigations and follow‐up will be carried out to evaluate the incidence rate of radiation pneumonitis and other side effects with the new treatment setup method.

It should be mentioned that in conventional supraclavicular irradiation, a small gantry tilt (e.g., 10°–15° moving away from the cord) with focused blocks may be used to reduce the radiation dose to the spinal cord for some patients.^(24)^ This technique cannot be combined with our new method because we have rotated both the couch and the gantry to reduce the dose to the lung tissues. However, one can achieve similar cord avoidance using the quarter‐field technique for the supraclavicular field, which will require a lateral couch shift. In our practice, we also limit our supraclavicular field to the side of the trachea and rotate the collimator 90° to use the MLC to form the lateral field edges. Overall, one has to compromise between cord avoidance and lung dose reduction and decide whether to use the conventional technique or the new method.

## V. CONCLUSIONS

Our new method does not alter the patient positioning for breast treatment and only requires rotating the couch to deliver a tilted supraclavicular field (vertical to the breast board) to maintain adequate CTV coverage and to spare more normal lung tissues. The results of this study demonstrated that our new method is effective, and the reduction of normal lung tissues is significant.
